# Macrophage Extracellular Traps in the Oral Mucosa: Autoimmune Disease and Platelet-Derived Epithelial Modulation

**DOI:** 10.3390/life16050751

**Published:** 2026-05-01

**Authors:** Stelvio Tonello, Nicole Vercellino, Davide D’Onghia, Marco Bagnati, Daniele Sola, Pier Paolo Sainaghi, Donato Colangelo

**Affiliations:** 1 Dipartimento di Medicina Traslazionale, Università del Piemonte Orientale, Via Solaroli 17, 28100 Novara, Italy; 2 Dipartimento di Scienze Teoriche e Applicate (DiSTA), Università eCAmpus, Via Isimbardi 10, 22060 Novedrate, Italy; 3 Initiative for RNA Medicine, Harvard Medical School, Boston, MA 02115, USA; 4 Clinical Biochemistry Laboratory, Azienda Ospedaliero-Universitaria Maggiore Della Carità di Novara, Corso Mazzini 18, 28100 Novara, Italy; 5 Laboratory of Metabolic Research, IRCCS Istituto Auxologico Italiano, Ospedale S. Giuseppe, 28824 Oggebbio, Italy; 6 Dipartimento di Scienze della Salute, Farmacologia, Scuola di Medicina, Università del Piemonte Orientale, Via Solaroli 17, 28100 Novara, Italy; donato.colangelo@med.uniupo.it

**Keywords:** macrophage extracellular traps (METs), ETs, autoimmune disease, oral mucosa, keratinocytes

## Abstract

Extracellular traps (ETs) are immune-derived chromatin networks initially described as antimicrobial barriers but increasingly recognized as modulators of tissue homeostasis and autoimmunity. The oral mucosa, constantly exposed to inflammatory stimuli, is particularly sensitive to ET-mediated remodeling (extracellular traps-mediated remodeling). In this study, we investigated how platelet-rich plasma (PRP), platelet-poor plasma (PPP), and washed platelets (WPT), widely used in regenerative medicine, influence ETosis in monocytes and macrophages, and how these ETs modulate the responses of primary buccal keratinocytes (pBMKs). ETs were induced in monocytes/macrophages using PRP, PPP, and WPT. pBMKs were exposed to ET-rich supernatants, and proliferation was monitored in real time through a live cell imaging system. ETs derived from PRP, PPP, and WPT did not induce either a statistically significant proliferation or morphological changes in buccal keratinocytes. These findings suggest that both platelet-derived products (PRP, PPP, WPT) and ETs play a crucial role in modulating epithelial biology, thus suggesting their possible role in chronic autoimmune diseases characterized by persistent inflammation and epithelial remodeling.

## 1. Introduction

Extracellular traps (ETs), including neutrophil extracellular traps (NETs) and monocyte/macrophage extracellular traps (METs), are extracellular chromatin networks released by immune cells in response to diverse stimuli. Originally described as antimicrobial barriers, ETs are increasingly recognized as modulators of tissue homeostasis, inflammation, and autoimmunity [1]. The underlying concept is that ETs not only neutralize pathogens but also actively modulate tissue microenvironments through signaling molecules, danger-associated molecular patterns, and interactions with resident stromal and epithelial cells [2]. In particular, ETs have been shown to influence epithelial and mesenchymal cell behavior, including keratinocyte proliferation, revealing a bidirectional interface between innate immunity and epithelial remodeling [3,4,5]. On one hand, this duality underscores a broader paradigm in which ETs participate in tissue repair and homeostasis; on the other hand, it exacerbates inflammatory damage, depending on the context and regulation of ET formation [6]. The oral mucosa represents a unique anatomic compartment in which ET-driven remodeling may have important consequences. Constant exposure to mechanical abrasion, microbiota, and environmental insults makes the oral surface highly responsive to innate immune cues. Growing evidence indicates that ETs, along with platelet-derived products, can shape epithelial and mesenchymal cell responses, thereby influencing wound healing trajectories and local inflammatory states within the oral cavity [6,7]. METs, generated by activated macrophages via a process termed METosis, share conceptual similarities with NETs but derive from a distinct leukocyte lineage. METs and NETs consist of extracellular chromatin decorated with histones and a range of antimicrobial proteins and proteases and can be triggered by stimuli that generate oxidative stress and engage autophagic signaling [8]. ETs, in general, can trap microbes, but they also serve as sources of inflammatory mediators and damage-associated molecular patterns (DAMPs) that influence neighboring cells and drive tissue remodeling. The METs and NETs, thus, represent a series of innate immune responses caused by different cellular inputs, leading to various consequences for inflammatory milieus, particularly within mucosal tissues, such as the oral cavity. In this framework, macrophage-derived cytokines, notably interleukin-1β (IL-1β), can direct the fate and function of adjacent stromal cells during wound healing [9]. IL-1β released in ET-rich environments can drive metabolic reprogramming in fibroblast progenitors, influencing proliferation, differentiation, and matrix remodeling. This macrophage–fibroblast crosstalk exemplifies how ET activity can translate innate immune signals into mesenchymal responses that shape healing outcomes and tissue architecture in mucosal sites [10]. Such interactions are especially relevant in the oral mucosa, where epithelial–mesenchymal communication regulates barrier integrity, repair, and remodeling in health and disease [11]. Regenerative strategies leveraging autologous platelet derivatives, namely, platelet-rich plasma (PRP), platelet-poor plasma (PPP), and washed platelets (WPT), have gained traction in dentistry and regenerative medicine for their bioactive cargo and pro-healing properties [11]. Notably, ET-rich supernatants derived from PRP/PPP can stimulate epithelial cell proliferation, implicating nucleic acid-sensing pathways and autophagy in mediating these effects [12]. Pharmacologic blockade of these pathways (e.g., through inhibitors of TLR9/cGAS–STING or autophagy) can negate the proliferative responses, underscoring the intricate balance between regenerative stimulation and inflammatory modulation in ET-rich milieus [13]. Conversely, PPP and WPT may differentially influence ET formation and downstream epithelial responses, with potential implications for both acute wound repair and chronic oral inflammation [14]. Taken together, these data establish platelet-derived components as modulators of ET dynamics and point to their therapeutic potential in optimizing tissue repair while restraining ET-mediated inflammatory responses in autoimmune environments [15]. Beyond the oral cavity, ETs have been implicated in systemic autoimmune processes and mucosal inflammatory conditions. NETs are abundant in systemic lupus erythematosus (SLE), where they provide autoantigens and amplify type I interferon signaling, linking ET biology to systemic autoimmunity and mucosal involvement [5,16]. This mechanistic nexus helps explain why oral ulcers and mucosal lesions frequently accompany systemic autoimmune diseases and why strategies that modulate ET activity may impact both local oral health and systemic disease activity [17,18]. Within the oral mucosa, ETs may participate in a dynamic epithelial–mesenchymal axis and mucosal immunity [19]. The oral cavity harbors abundant macrophage populations and pattern recognition receptors, providing a fertile milieu for ET formation and signaling. ETs can amplify local inflammation and drive barrier remodeling, potentially shaping disease trajectories in autoimmune oral conditions [19,20]. The interplay between ETs, keratinocytes, fibroblasts, and regenerative therapies suggests that ET biology could influence healing dynamics and chronic inflammatory remodeling in Sjögren’s syndrome, SLE-related oral lesions, pemphigus, mucous membrane pemphigoid, and related conditions [14]. While direct mechanistic studies in human oral diseases are evolving, the convergence of ET biology with mucosal immunity and epithelial–stromal crosstalk provides a plausible framework for understanding disease pathogenesis and identifying therapeutic targets in oral autoimmunity [21,22]. Nucleic acid sensing via TLR9 and the cytosolic cGAS–STING axis, together with autophagic processes, have emerged as pivotal mediators linking ETs to epithelial and fibroblast responses. Inhibiting these pathways can modulate both proliferative and inflammatory outcomes in oral epithelial cells, offering potential strategies to attenuate mucosal inflammation while preserving antimicrobial defense [23,24,25]. The translational implications of this study are multifaceted. First, targeting oxidative stress and autophagy pathways that drive ETosis may provide a strategy to attenuate chronic ET-mediated inflammation without compromising antimicrobial functions [26]. Second, ET components themselves (DNA fragments, histones, proteases) may be considered as biomarkers of mucosal immune activation in autoimmune oral diseases, enabling prognosis, patient stratification, and therapy monitoring through non-invasive sampling of saliva or gingival crevicular fluid [27]. The notion of a homeostatic yet tightly regulated ET milieu offers a compelling framework for future clinical research and therapeutic development in oral immunology and regenerative dentistry. The aim of this study was to identify the role of ETs produced by PRP, PPP, and WPT on the morphology and functional responses of human monocytes/macrophages in an in vitro buccal mucosa autoimmune disease model.

## 2. Materials and Methods

### 2.1. Platelet Fraction Preparation

Whole blood (10 mL) of 10 volunteer donors was collected into sodium citrate tubes (3.2%). Samples were centrifuged at 400× *g* for 10 min to separate plasma and cellular components. The top plasma fraction was allocated to two portions: one was kept as platelet-poor plasma (PPP), and the remaining plasma underwent a second centrifugation at 800× *g* for 10 min to pellet platelets, producing platelet-rich plasma (PRP). A third aliquot of plasma was used to obtain washed platelets (WPT) by washing the platelet pellet three times with PBS 1X and resuspending in the original plasma volume. Unless otherwise indicated, platelet preparations were carried out at room temperature and used on the day of preparation.

### 2.2. Monocyte/Macrophage Isolation

Blood from ten healthy donors collected in K2-EDTA/lithium/heparin tubes was diluted 1:1 with PBS 1X and separated using Lymphocyte Separation Medium 1077 (Merck KGaA, Darmstadt, Germany) according to the manufacturer’s protocol. Erythrocyte contamination was addressed by hypotonic lysis with a Red Blood Cell (RBC) lysis buffer when required. Monocytes/macrophages, prepared as previously described, were washed with sterile phosphate-buffered saline (PBS) 1X, counted, and resuspended at 5 × 10^4^ cells/mL in RPMI-1640 Medium (Merck KGaA, Darmstadt, Germany) without fetal bovine serum (FBS) and seeded onto glass microscope slides to allow adherence for 1 h at 37 °C. Non-adherent cells were removed by gentle aspiration [28,29].

### 2.3. ETosis Induction in Macrophages

Adherent macrophages were stimulated for 3 h with the stimuli PRP, PPP, and WPT, each adjusted to equivalent platelet content. Unstimulated wells served as controls. Lipopolysaccharide (LPS) (Merck KGaA, Darmstadt, Germany) and Phorbol 12-myristate 13-acetate (PMA) (Merck KGaA, Darmstadt, Germany), both at 100 nM, were used as positive controls for ETosis induction. ET release was characterized and quantified by confocal imaging.

### 2.4. ETosis Visualization (Confocal Microscopy)

For visualization, 5 × 10^3^ cells per well were seeded on poly-L-lysine-coated glass slides and treated with the indicated stimuli. After stimulation, cells were fixed overnight at 4 °C in PBS 1X containing 3.7% formaldehyde and 3% sucrose. Fixed cells were stained with SYTOX Green (Life Technologies Italia, Segrate, Italy) to detect extracellular DNA. Confocal microscopy was performed using a Leica DMIRE2 instrument (Leica, Wetzlar, Germany) with excitation at 488 nm (SYTOX Green) and appropriate emission filters. ET structures (ETs) were identified as extracellular chromatin NETs positive for SYTOX Green.

### 2.5. ETosis Quantification (Extracellular DNA Assay)

ETs were quantified following the protocol of Vong et al. [30]. Briefly, cells were plated at 1 × 10^5^ cells/well in Hanks’ Balanced Salt Solution (HBSS) 1X (Life Technologies Italia) and stimulated with PMA (100 nM) to induce ET formation, with Triton X-100 (0.5%) (Thermo Fisher Scientific, Waltham, MA, USA) as the positive control to obtain total DNA or left untreated as a negative control. After 2 h, DNase I (5 U per well) was added to all wells except the controls and Triton X-100 wells. After 45 min, SYTOX Green dye (Thermo Fisher Scientific, Waltham, MA, USA) was added (final concentration according to the manufacturer’s instructions) and plates were incubated for an additional 15 min. Fluorescence (excitation 504 nm, emission 523 nm) was measured using a plate reader (Victor X4, PerkinElmer, Shelton, CT, USA). Data are presented as relative units of fluorescence.

### 2.6. Keratinocyte Culture and Exposure to ETs

pBMKs were derived from the oral mucosa biopsies of three healthy donors, enzymatically dissociated (Trypsin 0.25%, 15 min at 37 °C), and cultured in keratinocyte serum-free medium supplemented with epidermal growth factor (EGF). ET-rich supernatants were collected by centrifugation of ETosis-induced cultures to remove cells, and the extracellular DNA content of supernatants was quantified (QuantiFluor^®^ ONE dsDNA System Promega, Madison, WI, USA). We seeded 5000 cells per well that were stimulated for 36 h with the different stimuli. Untreated pBMKs represent the control condition. Keratinocytes were exposed to ET-rich supernatants at a DNA-equivalent concentration of 100, 10, 1 and 0.1 ng/mL. For ET quantification in a pBMK context, extracellular DNA was quantified using the QuantiFluor^®^ ONE dsDNA System (Promega) following the manufacturer’s instructions for cellular stimulation.

### 2.7. Real-Time Proliferation Assay

Keratinocyte proliferation in response to PRP, PPP WPT and ETs was monitored with the IncuCyte^®^ SX5 Live-Cell Analysis System 2024B (Essen BioScience, Santa Clara, CA, USA). pBMKs were seeded at 5 × 10^3^ cells per well in 48-well plates. Images were captured every 3 h for 36 h, and confluence was analyzed automatically by the IncuCyte software version 2024B, with baseline normalization. Keratinocytes were stimulated with ETs at concentrations of 0, 0.1, 1, 10, and 100 ng/mL DNA-equivalents, and with PRP, PPP, and WPT at 10% in RPMI 1640 + 10% FBS as additional conditions.

### 2.8. Statistical Analysis

All experiments were conducted in triplicate and repeated independently where feasible. Data are presented as the mean ± standard deviation. Statistical significance was assessed by one-way analysis of variance (ANOVA) followed by Dunnett’s multiple comparison test. A *p*-value < 0.05 was considered statistically significant. All analyses were performed using GraphPad Prism version 7.0.

## 3. Results

**Figure 1:**
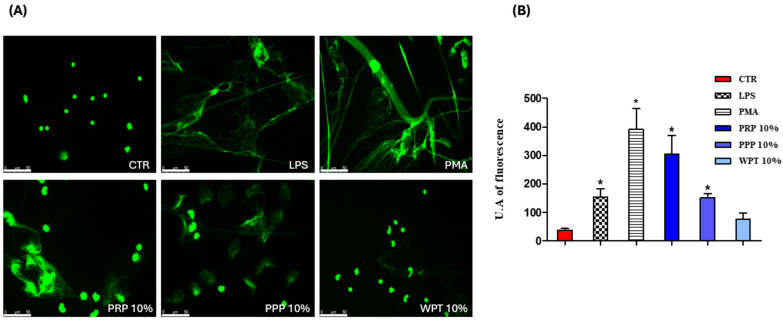
ET visualization and quantification by confocal microscopy in human monocyte-derived macrophages stimulated for three hours with platelet-rich plasma (PRP), platelet-poor plasma (PPP), or washed platelets (WPT). Unstimulated wells served as controls. LPS 100 ng/mL in RPMI 1640, and PMA (100 nM in RPMI 1640) were used as a positive control for ETosis. (**A**) Confocal visualization: The cells, seeded onto glass microscope slides and treated as described above, were stained for 15 min with SYTOX Green dye (Thermo Fisher Scientific, Waltham, MA, USA) and then fixed overnight at 4 °C in the dark using a 3.7% formaldehyde, 3% sucrose solution in PBS and visualized using a Leica DMIRE2 confocal microscope (Leica, Wetzlar, Germany) operating in fluorescence with an Ar/HeNe laser (488 nm). (**B**) Quantification of monocyte/macrophage extracellular traps: The fixed cells were stained with SYTOX Green to visualize monocytes/macrophages extracellular DNA (green). For each slide at least 3 different fields were acquired, and the DNA-associated fluorescence was evaluated using ImageJ software version 1.50f (National Institutes of Health, Bethesda, MD, USA). Results are expressed as arbitrary fluorescence units (mean ± SD; n = 10 independent healthy donors). Statistical significance was assessed by one-way analysis of variance (ANOVA) followed by Dunnett’s post hoc test (* = *p* < 0.05). Scale bar 50 µm. CTR = Control.

The presented results show, for the first time, whether platelet-derived fractions induce macrophage extracellular traps and, subsequently, they assess whether the same stimuli, including ETs, influence the morphology and proliferation of pBMKs. ET formation was evaluated qualitatively and quantitatively by confocal imaging, while keratinocyte behavior was monitored by live cell imaging over a 0- and 36-h window. In Figure 1A, confocal images related to monocyte/macrophage cells stimulated with PRP, PPP and WPT at a concentration of 10% in RPMI 10% FBS are presented. Figure 1B shows the quantification of monocyte/macrophage extracellular traps expressed in arbitrary units of fluorescence.

**Figure 2:**
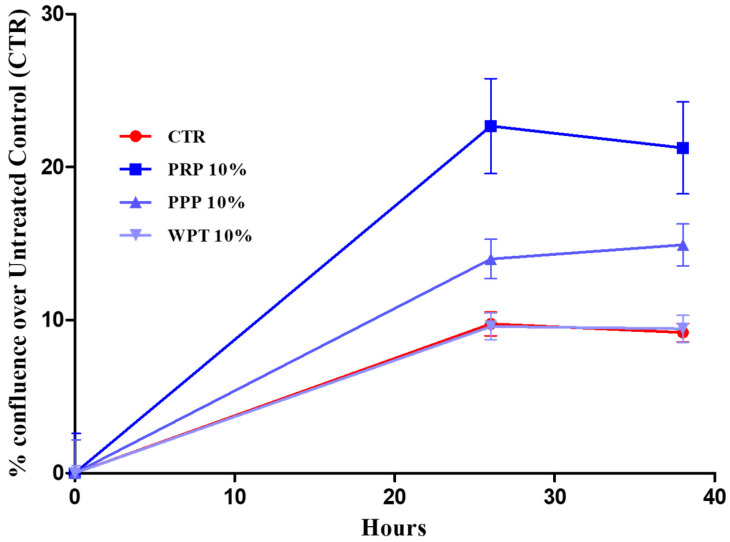
Effect of PRP, PPP and WPT on pBMK proliferation was monitored using the Incucyte^®^ SX5 Live-Cell Analysis System. Cells were seeded at 5 × 10^3^/well in 48-well plates, and images were acquired at 0, 24 and 36 h. Confluence was calculated automatically and normalized to baseline. pBMKs were seeded in RPMI 1640 10% FBS and stimulated with PRP, PPP and WPT 10% in RPMI 1640 0% FBS. No significant differences were reported. CTR = Control.

**Figure 3:**
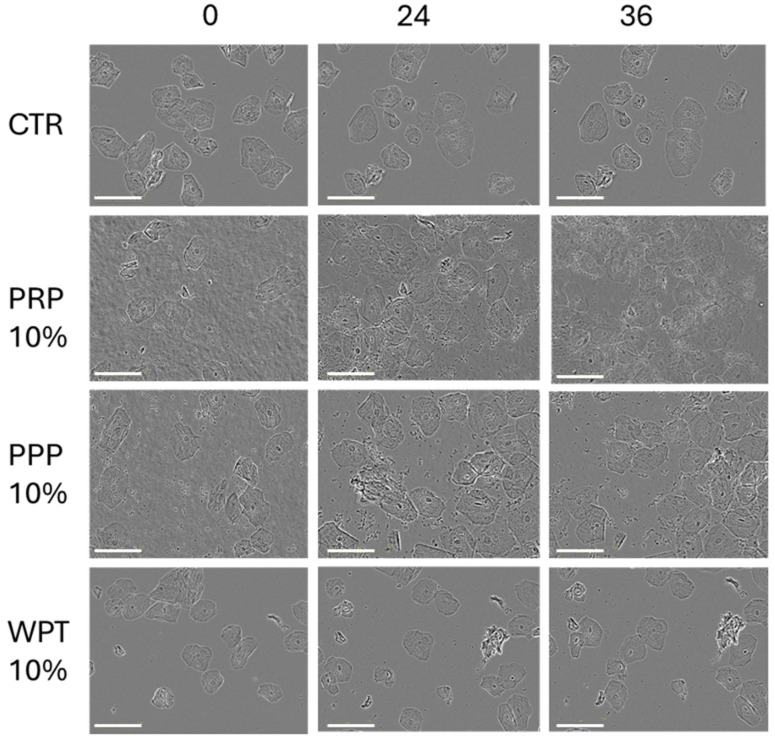
Images of pBMKs at different time points of 0, 24, 36 h stimulated with PRP 10%, PPP 10%, WPT 10% and control (CTR). Keratinocytes were seeded in RPMI 1640 0% FBS, with imaging at 0, 24, and 36 h. Confluence remained consistent with baseline and did not differ from corresponding controls at any time point. This result demonstrates that higher platelet-derived stimuli exposure does not translate into measurable changes in keratinocyte proliferation within 36 h in this experimental context. Scale bar = 100 µm. CTR = Control.

Having established ET induction in macrophages, we evaluated whether the same platelet-derived stimuli, and the associated extracellular traps, influence the morphology and growth of pBMKs under comparable experimental conditions. Figure 2 and Figure 3 show how pBMKs respond to platelet-derived stimuli in a live-cell imaging setup, focusing on morphology and proliferation over time. Across the 0- and 36-h intervals, no overt morphological changes were observed in pBMKs under any platelet-derived stimulus. Figure 2 shows the keratinocyte proliferation monitored using the Incucyte^®^ SX5 Live-Cell Analysis System and the results are expressed as a percentage of confluence.

**Figure 4:**
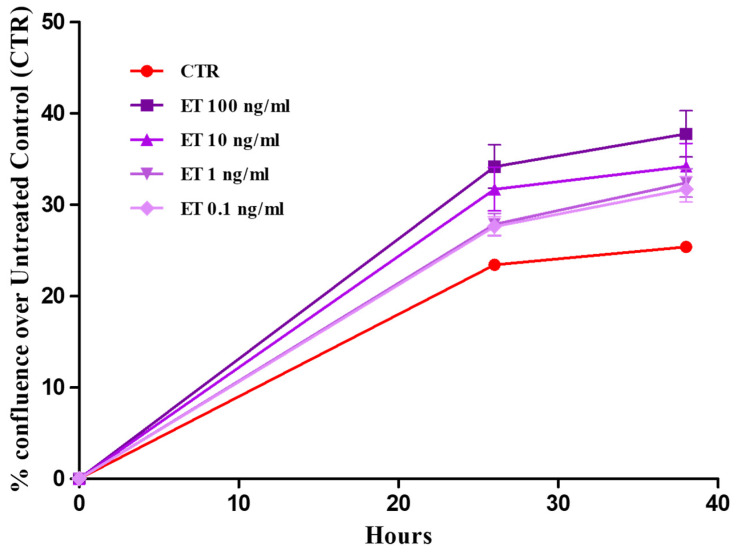
Effect of increasing amounts of ETs on pBMK proliferation was monitored using the Incucyte^®^ SX5 Live-Cell Analysis System. Cells were seeded at 5 × 10^3^/well in 48-well plates, and images were acquired at 0, 24 and 36 h. Confluence was calculated automatically and normalized to baseline. pBMKs were seeded in RPMI 1640 10% FBS and stimulated with ETs in RPMI 1640 0% FBS. CTR = Control.

**Figure 5:**
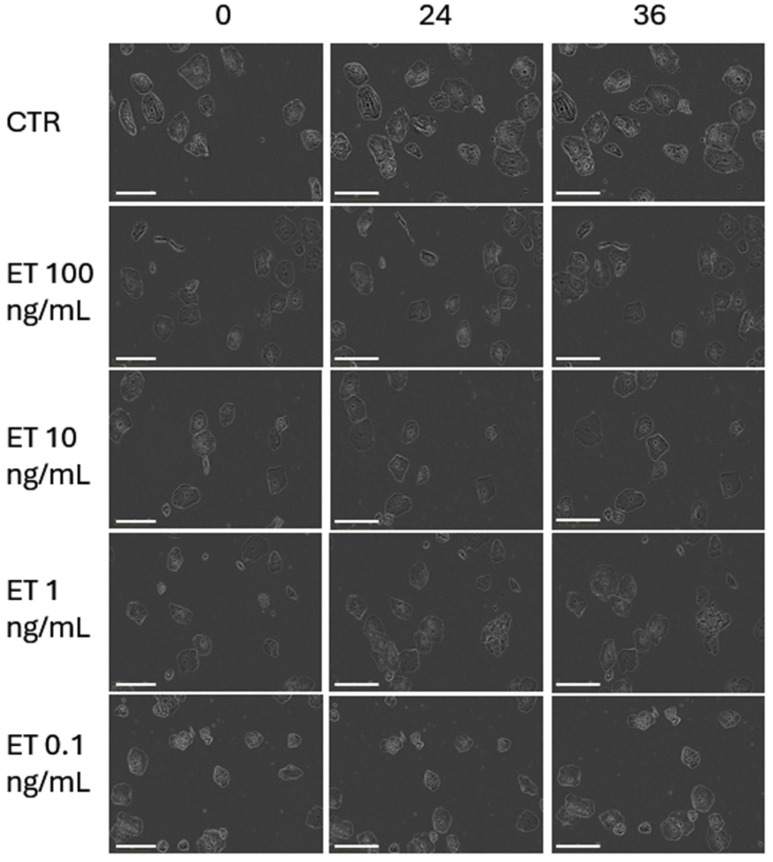
Effect of increasing amounts of ETs on pBMK proliferation was monitored using the Incucyte^®^ SX5 Live-Cell Analysis System. Cells were seeded at 5 × 10^3^/well in 48-well plates, and images were acquired at 0, 24 and 36 h. Confluence was calculated automatically and normalized to baseline. pBMKs were seeded in RPMI 1640 10% FBS and stimulated with ETs in RPMI 1640 0% FBS. Scale bar = 100 µm. CTR = Control.

When exposed to ET-rich supernatants, primary buccal keratinocytes exhibited a not statistically significant proliferative response. The results of the proliferation analysis are presented in Figure 4 and expressed as a percentage of confluence. Figure 5 presents the images of pBMKs at intervals of 0, 24 and 36 h.

In Figure 5, we can appreciate the effect of different concentrations of ETs on pBMKs. The images were taken automatically from Real-time Incucyte^®^ SX5 imaging technology. Keratinocyte proliferation remained unchanged relative to baseline across all doses and time points, supporting the fact that the short-term growth dynamics of pBMKs are resistant to ET exposure under these culture conditions.

## 4. Discussion

The findings presented here offer a mechanistic framework for understanding how platelet-driven signals interface with mucosal immunity in the oral cavity, a region constantly exposed to microbial challenges, mechanical stress, and autoimmune manifestations in many patients [31,32]. In a controlled in vitro setting, platelet-derived fractions such as platelet-rich plasma (PRP), platelet-poor plasma (PPP), and washed platelets (WPT) drive the formation of METs (although WPT not at a significant level, *p* = 0.07), a chromatin-based antimicrobial mechanism that consolidates innate defense at sites of barrier disruption. By contrast, the same stimuli, including ETs, fail to provoke detectable short-term changes in the morphology or proliferation of pBMKs within a 0- and 36-h window. This cell-type-specific divergence, and a strong ET response in macrophages without a concomitant keratinocyte proliferative reaction, provides a dynamic lens through which to view the mucosal immunopathology characteristic of autoimmune diseases affecting the oral mucosa, such as pemphigus vulgaris (PV), mucous membrane pemphigoid (MMP), oral lichen planus (OLP), Sjögren’s syndrome with oral involvement, and Behçet’s disease [33]. Platelets have emerged as active regulators of mucosal immunity rather than mere hemostatic effectors [34]. They release a diverse cargo such as chemokines, growth factors, microparticles, and nucleic acids that contain vesicles, which can engage macrophage receptors and reshape signaling toward chromatin decondensation and extracellular trap formation. ETs, comprising chromatin networks decorated with antimicrobial proteins, can immobilize pathogens but may also propagate local inflammation through histone-mediated cytotoxicity and proteolytic enzymes [35]. Nucleic acid sensing via TLR9 and the cytosolic cGAS–STING axis, together with autophagic processes, have emerged as pivotal mediators linking ETs to epithelial and fibroblast responses. Inhibiting these pathways can modulate both proliferative and inflammatory outcomes in oral epithelial cells, offering potential strategies to attenuate mucosal inflammation while preserving antimicrobial defense [4,5,13,21]. In our experiments, the concordance between qualitative imaging and quantitative metrics across distinct platelet-derived fractions implies that a shared platelet-associated cue suffices to allow ET assembly in human macrophages. Mechanistically, ET induction likely reflects a convergence of soluble mediators and platelet-derived macrovesicles modulating pattern recognition receptor (PRR) signaling, reactive oxygen species (ROS) generation, and chromatin remodeling [23]. Some authors demonstrated by using stimuli like PMA that MAPK and Ras-Raf-ERK signaling pathways significantly increase extracellular trap formation and release, and ROS production [24]. While our study did not examine these pathways in detail, the consistent ET response across PRP, PPP, and WPT aligns with a model in which multiple platelet-derived cues collectively contribute to ET generation, with ROS production and PAD4 activity representing plausible downstream mediators that warrant focused mechanistic exploration [5,36]. In sharp contrast to ET induction in macrophages, pBMKs displayed notable strength to the platelet-derived cues tested. Across the 0- and 36-h window, there were no significant morphological changes or proliferative shifts in keratinocytes in response to PRP, PPP, WPT, or ETs. This observation suggests a degree of early compartmentalization in mucosal responses: macrophage ET activation may function as an initial innate defense that does not automatically translate into rapid epithelial expansion, at least in monoculture systems [37,38,39]. Placing these results in the context of autoimmune diseases affecting the oral cavity generates several testable hypotheses with potential clinical relevance. An ET-rich milieu produced by platelet-driven macrophage activation could supply additional DAMPs and autoantigen material, potentially promoting epitope spreading or enhancing autoreactive immune cell activation [40,41]. Although our study did not measure autoantibody generation or antigen presentation directly, the ET-associated materials generated by platelet cues offer a plausible link between innate and adaptive autoimmunity in the oral mucosa [42]. Inflammatory mucosal states such as OLP and Behçet’s disease may also be influenced by ET-derived DAMPs, which could augment immune cell recruitment and cytokine networks, potentially sustain chronic inflammation or delay healing [43,44]. The core strength of this study lies in the convergent evidence that ETs are induced by diverse platelet-derived fractions, supported by both qualitative imaging and quantitative metrics across independent donors. These results also pave the way for future investigations that could bridge in vitro observations to clinical outcomes. Expanded epithelial readouts such as keratinocyte migration, barrier integrity (e.g., transepithelial electrical resistance), differentiation marker expression, and cytokine/antimicrobial peptide profiles would provide a richer view of epithelial responsiveness in autoimmune mucosa, particularly when studied in co-culture or organotypic models that better approximate the oral mucosa’s architecture and signaling milieu [45,46,47]. Integrating patient-derived materials, including autoantibody sera from PV/MMP and oral microbiota, could validate ET-related biomarkers and identify patient subgroups most likely to benefit from ET-targeted therapies. The present work relies on an in vitro monoculture system for keratinocytes and a 0–36-h observation window, which may not capture later-stage interactions or the full multicellular complexity of the oral mucosa. Donor heterogeneity and tissue context remain important considerations for extrapolating to human disease [48]. Nonetheless, the observed ET induction in macrophages, together with the lack of early keratinocyte proliferation, establishes a focused, hypothesis-generating framework that can be further explored using organotypic models, co-cultures, and patient-derived materials to more accurately recapitulate autoimmune oral diseases [49].

## 5. Conclusions

In conclusion, these findings anchor a mechanistic framework linking platelet–macrophage ET biology to the pathophysiology of autoimmune diseases affecting the oral cavity. ET responses to platelet-derived cues underscore the active involvement of platelets in shaping innate immune effector functions within mucosal tissues, with potential consequences for antimicrobial defense, tissue injury, and repair during autoimmune flares.

## Data Availability

The original data presented in the study are openly available in Public archive at https://www.uniupo.it/en#.

## Abbreviations

The following abbreviations are used in this manuscript:

cGAS-STING	Cyclic GMP-AMP synthase/Stimulator of Interferon Genes
DAMPs	Damage-Associated Molecular Patterns
ERK	Extracellular Signal-Regulated Kinase
ETs	Extracellular Traps
MAPK	Mitogen-Activated Protein Kinase
METs	Monocyte/macrophage Extracellular Traps
MMP	Mucous Membrane Pemphigoid
NETs	Neutrophile Extracellular Traps
OLP	Oral Lichen Planus
pBMKs	primary Buccal Mucosal Keratinocytes
PPP	Platelet-Poor Plasma
PRP	Platelet-Rich Plasma
PV	Pemphigus Vulgaris
Raf	Rapidly accelerated fibroblast–serine/threonine kinase
Ras	Rat sarcoma—small GTPase protein
TLR9	Toll-Like Receptor 9
WPT	Washed Platelet
